# Funding Trends in Japan Agency for Medical Research and Development (AMED): Focus on Psychiatry

**DOI:** 10.31662/jmaj.2024-0391

**Published:** 2025-04-04

**Authors:** Yuji Yamada, Megumi Haga, Yutaka Matsuoka

**Affiliations:** 1 Division of Data Sharing and Medical Art, Department of Health and Clinical Data, Japan Agency for Medical Research and Development, Tokyo, Japan; 2 Division of Data Utilization, Department of Health and Clinical Data, Japan Agency for Medical Research and Development, Tokyo, Japan; 3 National Cancer Center Institute for Cancer Control, Tokyo, Japan

**Keywords:** psychiatry, research and development (R&D), funding agency, medical device, digital mental health, pharmaceuticals

## Abstract

The Japan Agency for Medical Research and Development (AMED) was established in April 2015 as a funding agency for medical research and development. AMED has been striving to ensure the provision of state-of-the-art medical services and the advancement of a society characterized by health and longevity. Furthermore, AMED facilitates the seamless integration of research projects, spanning the spectrum from basic to applied research and practical applications. The current article presents an overview of the trends observed in awarded projects related to psychiatric disorders. Consequently, there was a considerable rise in the number of projects pertaining to medical devices, particularly within the domain of digital mental health. It is anticipated that an increased number of social implementation studies will obtain regulatory approval under the Pharmaceutical and Medical Device Act.

Psychiatric disorders are among the most severe medical conditions, exerting a profound impact on the patient’s life course. As indicated by the Global Burden of Disease study, these disorders represent the second leading cause of years lived with disability (YLDs) worldwide, with a notable increase in their disease burden ^(1)^. Depressive disorders were the second highest cause of YLDs in 2021 (56.3 million [95% confidence interval 39.3-76.5] YLDs), representing a 36.5% increase from 2010. Anxiety disorders were the sixth highest cause of YLDs (42.5 million [29.4-57.7] YLDs) ^(1), (2)^. Psychiatric disorders not only impair social functions but also increase the risk of developing physical diseases. Indeed, patients with psychiatric disorders have a shorter life expectancy than the general population, with a difference of 14.6 years ^(3)^. Consequently, it is very important that we address the issue of conquering psychiatric disorders.

The field of Research and Development (R&D) for psychiatric disorders is facing significant challenges. From 2011 to 2021, the Food and Drug Administration approved 135 new drugs in the field of oncology and 50 new drugs in neurology, whereas only 12 new drugs were approved in psychiatry ^(4)^. This discrepancy can be attributed to the relatively low success rate of drug development in the psychiatric field, which is attributable to a lack of understanding of the pathophysiology of psychiatric disorders and the underdevelopment of biomarkers that reflect therapeutic efficacy ^(5)^. As a result, the development of new treatments for psychiatric disorders has been stagnant ^(6)^.

The stagnation of R&D has led to a decline in funding, creating a vicious cycle. The low success rate in drug development for psychiatric disorders has resulted in many pharmaceutical companies being unable to recoup their investment. Furthermore, the high placebo response rate has the effect of increasing the sample size of clinical trials, which also places a significant burden on these companies ^(7)^. As a result, pharmaceutical companies such as Pfizer and AstraZeneca have largely discontinued the development of psychiatric drugs ^(6)^. These circumstances have made the R&D of new treatments increasingly challenging in this field.

In the field of psychiatry, the role of funding agencies is of paramount importance with regard to the development of innovative therapeutic modalities. Moreover, sustained and substantial investment in translational research is essential to establish the foundation for future treatment development. In light of these considerations, it is anticipated that public institutions and charitable funders will assume the responsibility of providing financial support. While charitable funders, such as Cancer Research UK and the Michael J. Fox Foundation, play a significant role in oncology and neurology, respectively, no such organizations were identified in psychiatry ^(6)^. It is therefore essential that public institutions make significant, long-term investments in research to overcome the current stagnation in the field of psychiatry.

This article indicates the role of the Japan Agency for Medical Research and Development (AMED) in providing funding. This article reviews the funding trends of the AMED in the field of psychiatry and discusses the prospects for innovative R&D in Japan.

The AMED was established in April 2015 as a funding agency with the objective of providing financial support for medical R&D activities, with the ultimate goal of enhancing the research environment in Japan. Furthermore, AMED seeks to facilitate the establishment of new industries in a comprehensive and systematic manner. These measures are to be implemented by the government in accordance with the Act to Promote Healthcare and Medical Strategy, which was promulgated in May 2014. The AMED assumes a command post function for R&D in implementing measures such as the “healthcare and medical strategy” and “plan for promotion of medical research and development,” which are established by the headquarters for healthcare policy in the cabinet ^(8)^. Prior to this, the respective ministries, namely the Ministry of Education, Culture, Sports, Science and Technology (MEXT), the Ministry of Health, Labor and Welfare (MHLW), and the Ministry of Economy, Trade and Industry (METI), had been conducting independent R&D activities in the medical field. Consequently, AMED integrated these R&D activities to establish a system that facilitated a seamless transition from basic research to practical application and ensured the execution of high-quality clinical research and trials on an international level. This objective was accomplished by consolidating R&D budgets from the MEXT, MHLW, and METI (Figure 1) ^(9)^.

**Figure 1.**
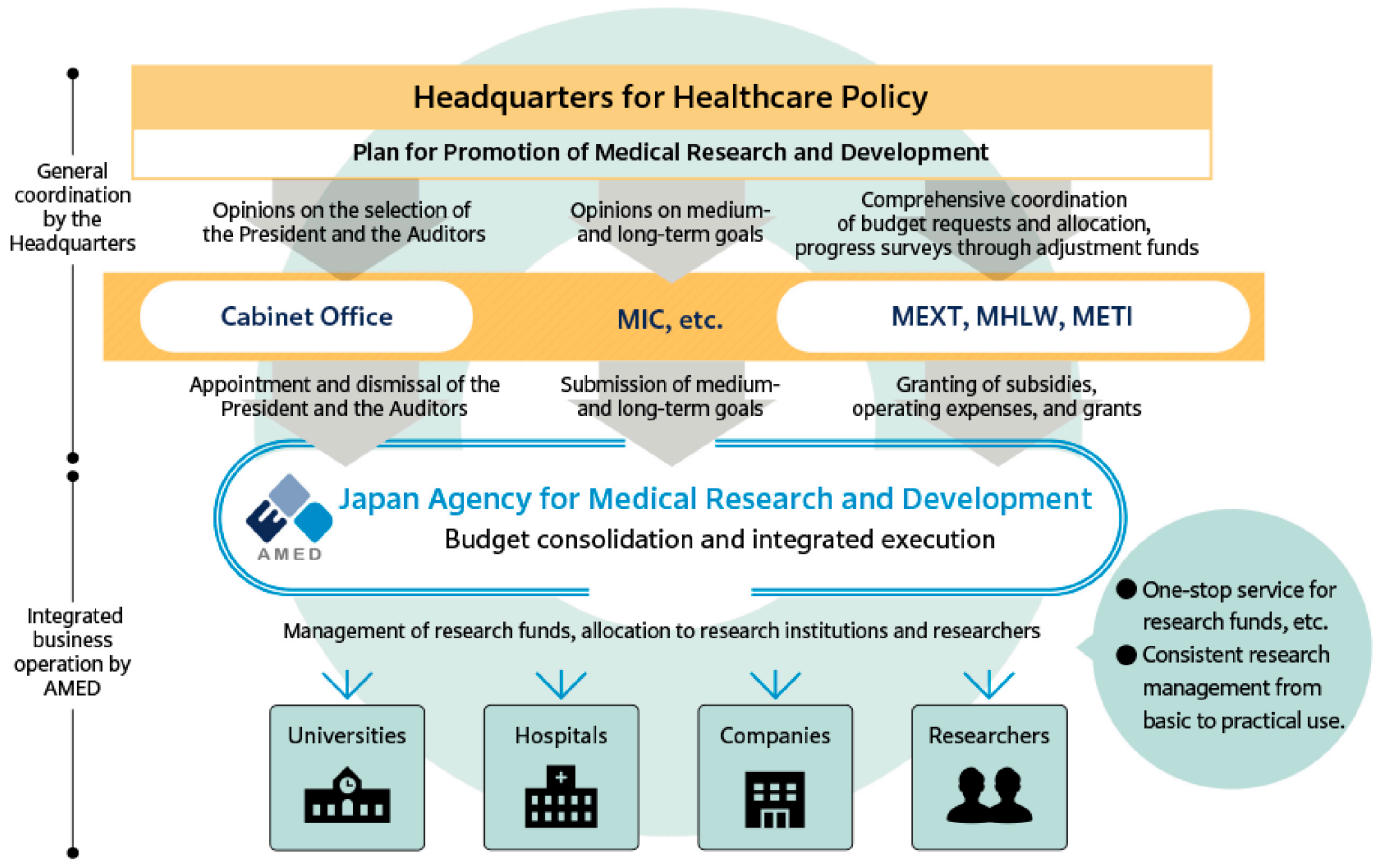
Positioning of AMED (adapted with permission from Guide to AMED) ^(9)^. AMED is an organization that plays a pivotal role in the field of medical R&D by integrating the R&D conducted independently by MEXT, MHLW, and METI. AMED: Japan Agency for Medical Research and Development; METI: Ministry of Economy, Trade and Industry. MEXT: Ministry of Education, Culture, Sports, Science, and Technology; MHLW: Ministry of Health, Labor, and Welfare; MIC: Ministry in charge; R&D: Research and Development.

In its second medium- to long-term plan (fiscal year [FY]2020-2024), AMED has established a series of integrated projects. The projects concentrate on six modalities (technologies/methods) and facilitate R&D in the medical field, from fundamental to practical applications, in an integrated manner under the direction of a program director (PD) in each project. Moreover, disease area coordinators with considerable experience in seven major disease areas are appointed across the integrated projects to ensure the effective application of the medical technologies developed in each project to a range of diseases. One such disease area is that of mental and neurological disorders (Figure 2) ^(9)^.

**Figure 2.**
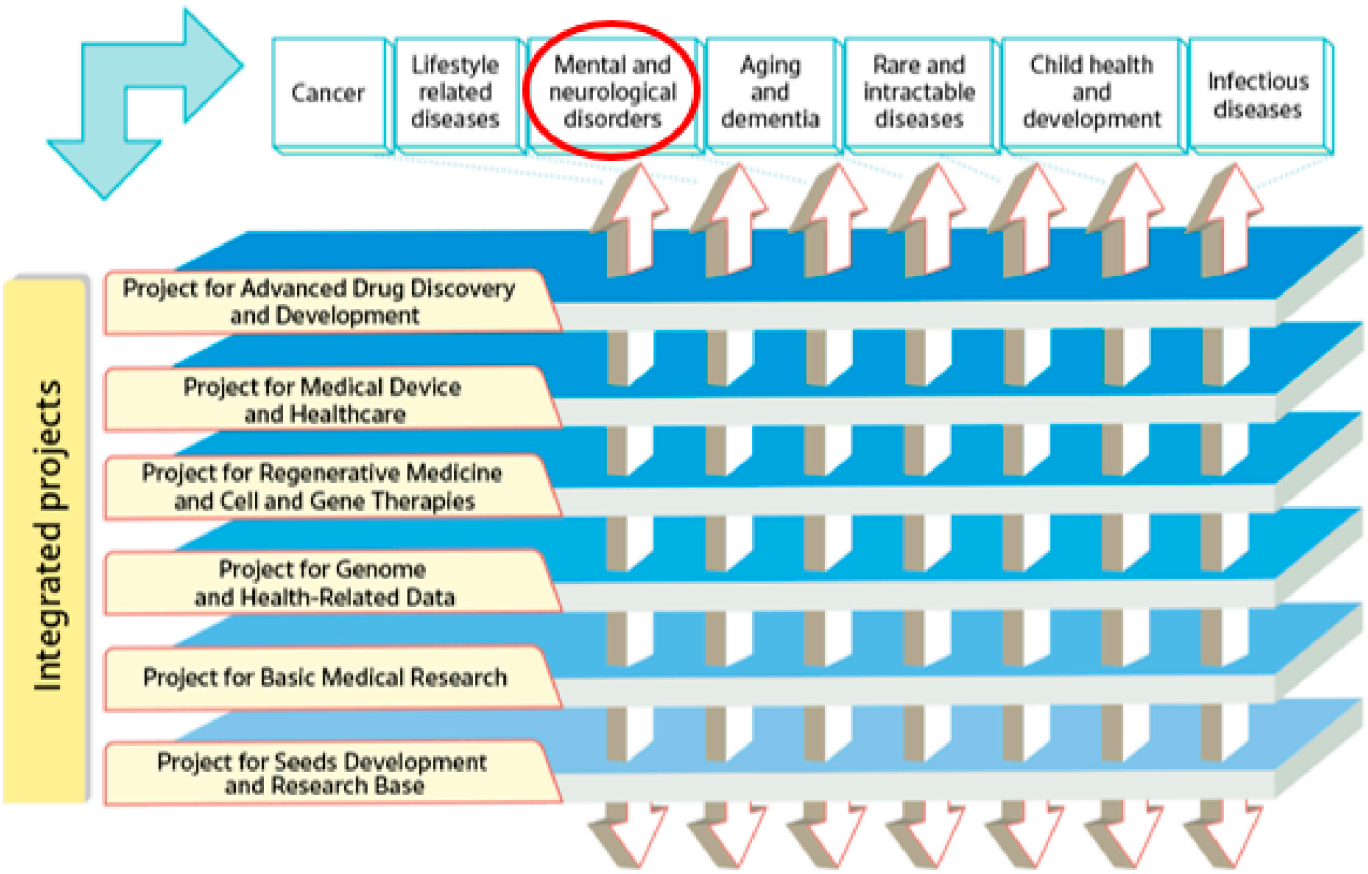
Six “Integrated Projects” centered on modalities (adapted with permission from Guide to AMED) ^(9)^. AMED has established six “Integrated Projects” centered on modalities. These projects are coordinated with the projects of related ministries and agencies under the PD. Researches in specific disease areas are promoted across the integrated projects so that it can be managed flexibly by the coordinators of each disease area. AMED: Japan Agency for Medical Research and Development; PD: program director.

For each integrated project, proposals are solicited from a diverse cohort of researchers and institutions, including universities, research institutes, and companies. The implementers are selected through a process of evaluation and selection. To evaluate and manage R&D projects, program supervisors (PS) and program officers (POs) with a high level of knowledge in the relevant research field are appointed. The PD, PS, and PO work together to gain an understanding of the issues pertaining to the integrated project, to manage them effectively, and to promote collaboration among the various projects. Furthermore, they evaluate and identify exceptional R&D proposals and consistently succeed in linking the results of basic science to clinical research.

In the initial medium- to long-term plan (FY2015-2019), AMED established the framework for funding methods and trends. The number of applications to the AMED fund was approximately 3,700-3,900 per year, with an approximately 18%-22% success rate ^(10)^. The number of awarded projects increased from 2,089 in 2015 to 2,593 over the first five-year term. This figure included projects that were adopted by ministries, such as MEXT and MHLW, which were responsible for these projects prior to the establishment of AMED. Subsequently, these projects were transferred to AMED after 2015. The amount of R&D funding remained at approximately 130 billion Japanese yen (JPY) per year (Supplementary Figure 1) ^(10)^. The median research duration for newly awarded projects was 2 years and 9 months, with the majority of them falling within the 2-3 year and 3-4 year ranges (Supplementary Figure 2) ^(10)^. Universities constituted the largest proportion of research institutions that allocated R&D funds, representing approximately 60% of the total. The next largest groups were national centers and companies, which constituted approximately 20% and 12% of the total, respectively (Supplementary Figure 3) ^(10)^. With regard to the allocation of R&D funds according to the International Classification of Diseases, 10th Revision, the majority of projects were not focused on a specific disease category. The “not applicable” category encompassed technologies and data infrastructure that could be applied to a range of diseases. The next most prevalent category was neoplasms and infectious diseases. The proportion of funding allocated to psychiatric disorders remained at 3%-4% of the total budget (Figure 3) ^(10)^. These trends were consistently observed throughout the initial medium- to long-term plan.

**Figure 3.**
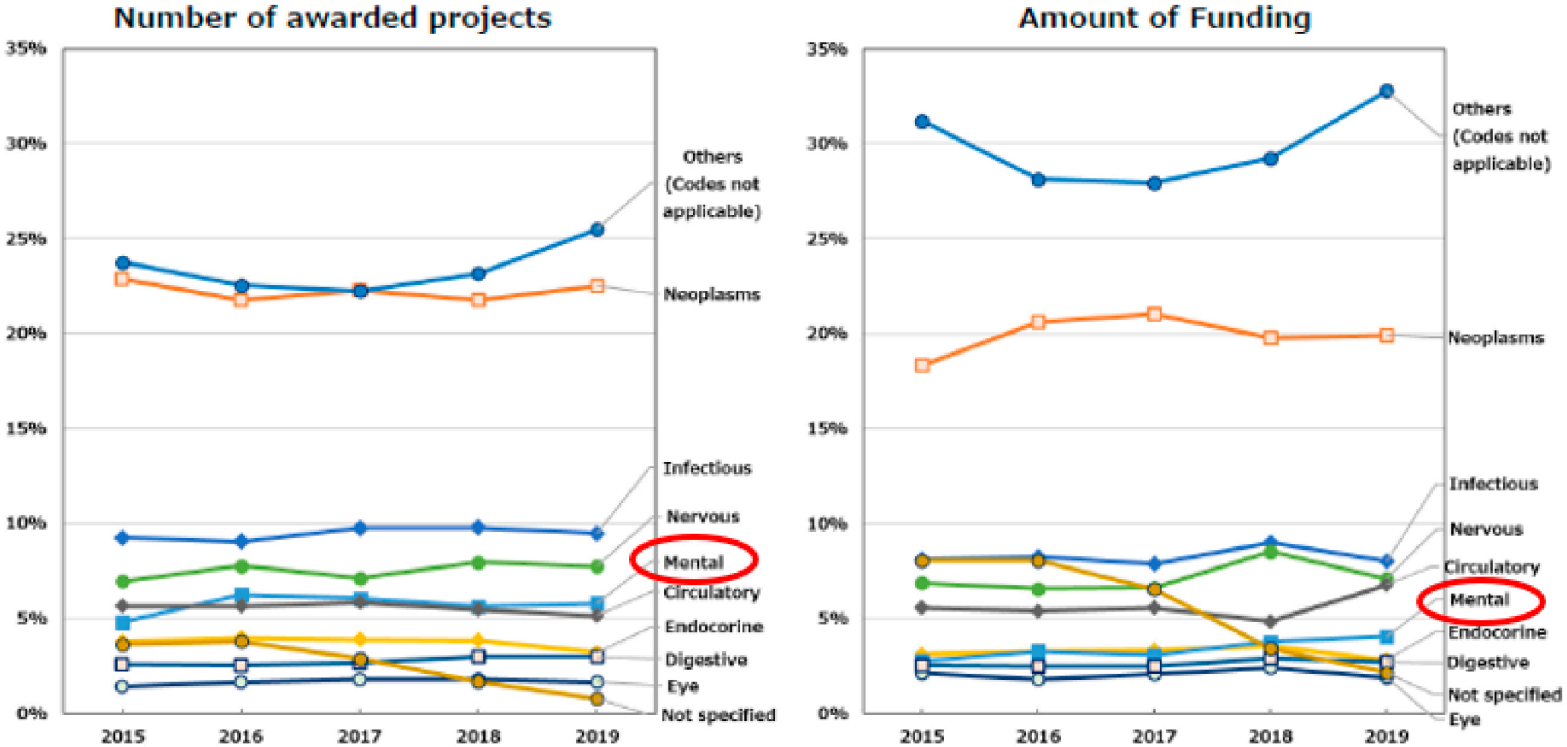
Trends in disease categories by ICD-10 in FY2015-2019 (adapted with permission from AMED data book: first medium- to long-term plan) ^(10)^. FY: fiscal year; ICD-10: International Classification of Diseases, 10th Revision.

The second medium- to long-term plan (FY2020-2024) of the AMED is distinguished by its emphasis on the advancement of R&D in the context of the COVID-19 pandemic. In particular, the number of applications and awarded projects increased in FY2020 as a consequence of supplementary budgets for R&D related to COVID-19 (Figure 4) ^(11)^. Consequently, following FY2020, R&D funding for new projects increased considerably in comparison to that allocated to continued projects. With regard to the distribution of R&D funds, the greatest number of projects in FY2022 were situated within the 10 million to 25 million JPY range, followed by those within the 25 million to 50 million JPY range (Figure 5) ^(11)^. Furthermore, AMED’s second medium- to long-term plan outlined integrated projects based on six modalities (technologies/methods). The integrated projects included the following areas of research: (1) advanced drug discovery and development, (2) medical devices and healthcare, (3) regenerative/cellular medicine and gene therapies, (4) genome and health-related data, (5) basic medical research, and (6) seeds development and research base (Figure 2). In FY2022, the integrated project for advanced drug discovery and development had the largest number of awarded projects and R&D funding (Figure 6) ^(11)^. This funding trend for pharmaceutical projects continued to prevail throughout the second medium- to long-term plan.

**Figure 4.**
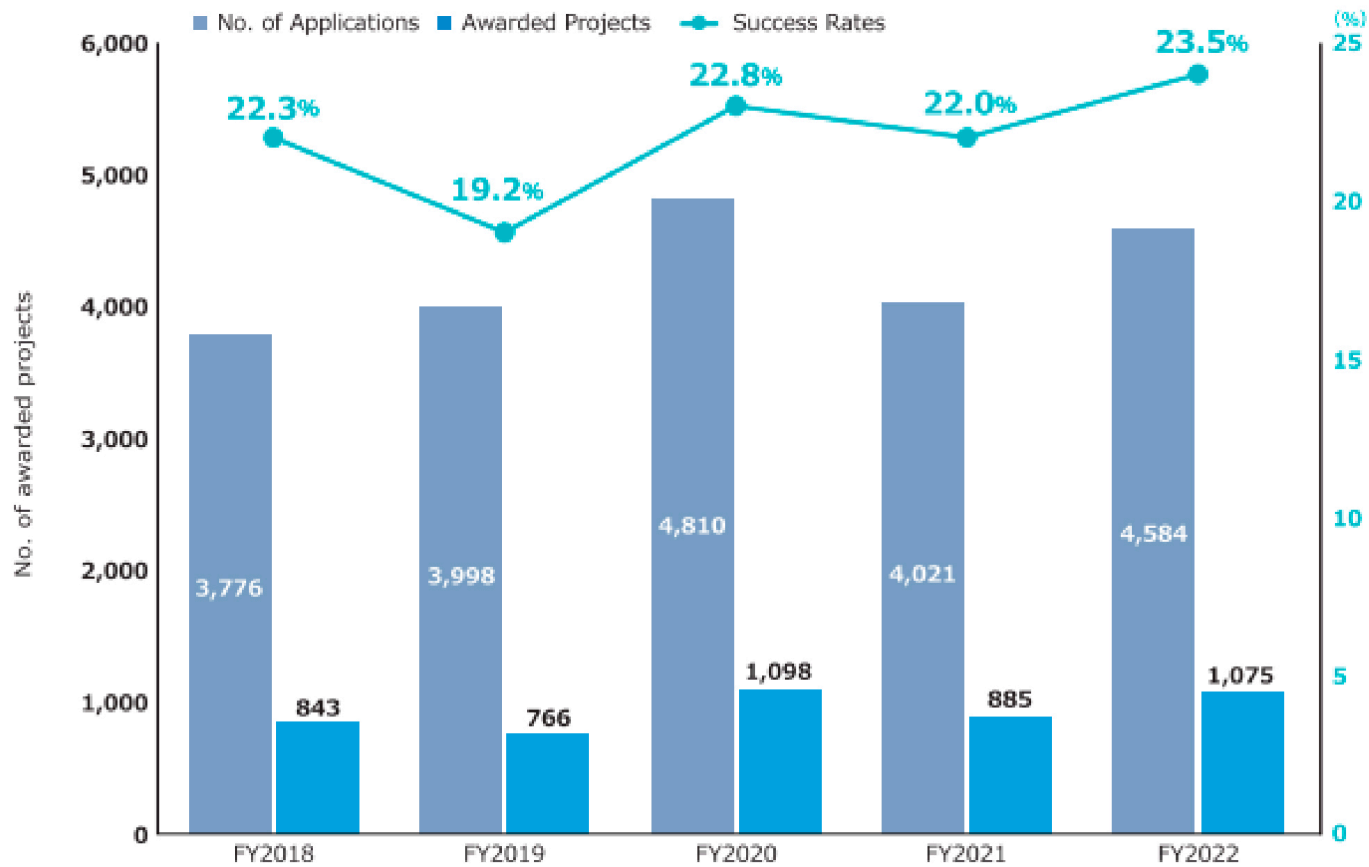
Number of applications, awarded projects, and success rates in FY2018-2022 (adapted with permission from AMED DataBook 2022) ^(11)^. The success rate is defined as the ratio of the number of all adopted proposals to the number of all applications received for each FY. This figure is calculated on an annual basis using publicly available information from AMED regarding open calls for proposals (as of October 2023). AMED: Japan Agency for Medical Research and Development; FY: fiscal year.

**Figure 5.**
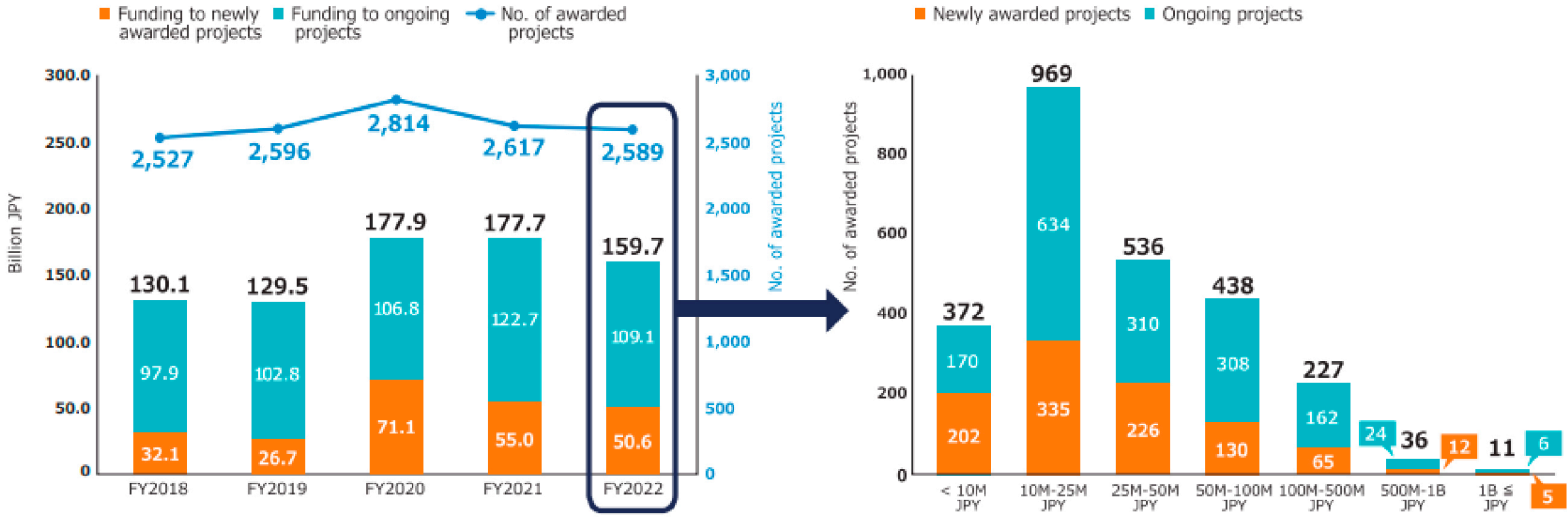
(Left) Number of awarded projects and amount of funding, (right) distribution of amount of funding of awarded projects in FY2022 (adapted with permission from AMED DataBook 2022) ^(11)^. This figure is derived from AMED data (as of October 2023). AMED: Japan Agency for Medical Research and Development; FY: fiscal year; JPY: Japanese yen.

**Figure 6.**
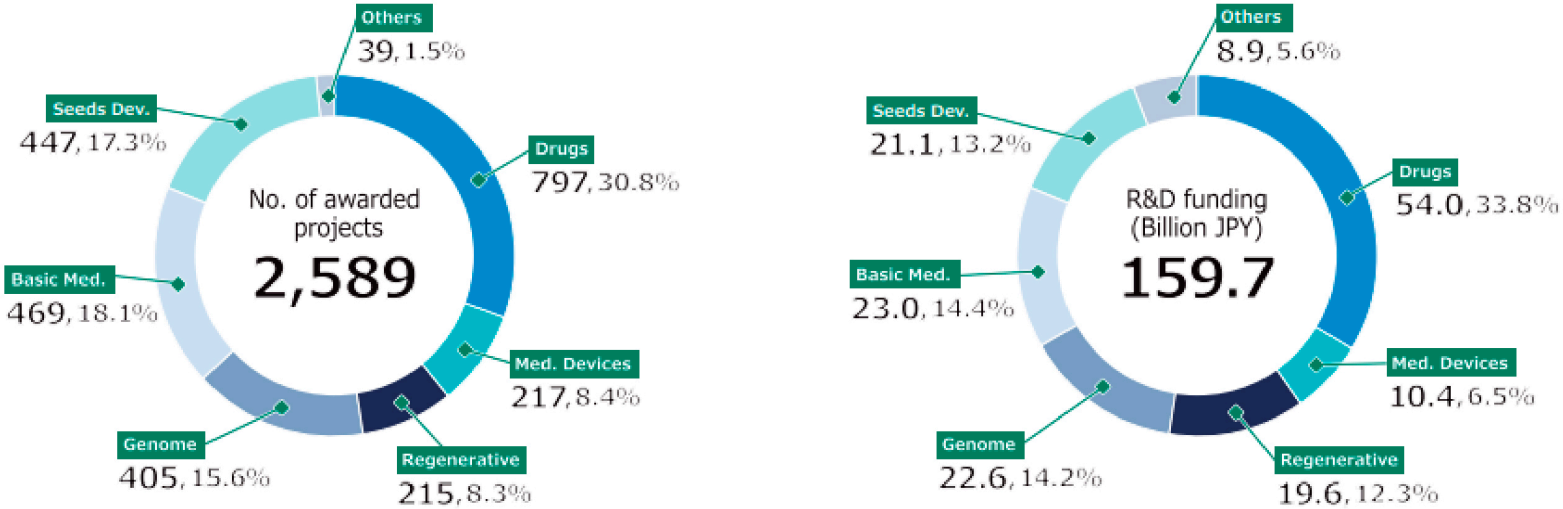
(Left) Number of awarded projects in FY2022, (right) R&D funding by integrated project in FY2022 (adapted with permission from AMED DataBook 2022) ^(11)^. This figure is derived from AMED data (as of October 2023). AMED: Japan Agency for Medical Research and Development; Basic Med.: projects for basic medical research; Drugs: projects for advanced drug discovery and development; FY: fiscal year; Genome: projects for genome and health-related data; JPY: Japanese yen; Med. Devices: projects for medical devices and healthcare; R&D: Research and Development; Regenerative: projects for regenerative/cellular medicine and gene therapies; Seeds Dev.: projects for seeds development and research base.

A review of newly adopted projects revealed that the majority of principal investigators (PIs) were male, whereas female PIs constituted only approximately 9%-10% of the total. With regard to the mean age of PIs, males were approximately 50 years old, while females were younger than males, with a mean age of 46-47 years (Figure 7) ^(11)^. In FY2022, the largest number of male PIs were between the ages of 40 and 44 (18% of the total number of male PIs), followed by those between the ages of 45 and 49, and 55 and 59. Fourteen percent of male PIs were under the age of 40. In contrast, the largest number of female PIs were between 45 and 49 years old (26% of all female PIs), followed by those between 40 and 44 years old. Furthermore, 20% of female PIs were under the age of 40 (Supplementary Figure 4) ^(11)^. It is expected that the number of female PIs and young PIs will further increase, contributing to the diversification and revitalization of AMED’s R&D activities.

**Figure 7.**
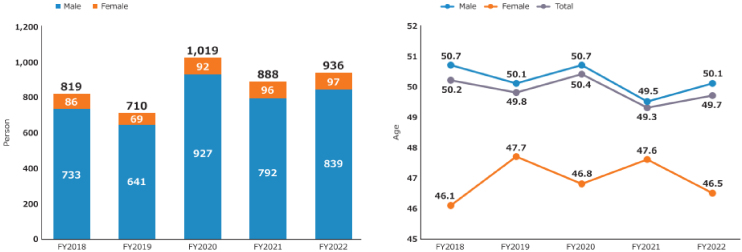
(Left) Trends in number of PIs by gender in FY2018-2022, (right) trends in average age of all PIs by gender in FY2018-2022 (adapted with permission from AMED DataBook 2022) ^(11)^. The number of PIs represents the total number of newly awarded projects in each FY. The age of each PI is calculated as of the commencement of the FY in which the research project was initiated, based on their date of birth. This figure is derived from the Cross-ministerial R&D Management System (e-Rad) (as of October 2023). FY: fiscal year; PI: principal investigator; R&D, Research and Development.

The R&D of AMED in the field of medical devices has been actively promoted in the context of psychiatry. To facilitate the delivery of R&D results to patients in Japan, it is necessary to obtain regulatory approval from the Pharmaceuticals and Medical Devices Agency. In light of the aforementioned considerations, the R&D trends are evaluated in accordance with the classification outlined in the Act on Securing Quality, Efficacy, and Safety of Products Including Pharmaceuticals and Medical Devices (Pharmaceutical and Medical Device Act). In the FY2020-2022, approximately 8%-9% of awarded projects included psychiatric disorders as the primary or secondary target diseases (Figure 8). The proportion of pharmaceutical projects pertaining to psychiatry remained at approximately 6%-8% of the total number of pharmaceutical projects throughout FY2020-2022. The major target conditions were dementia and developmental disorders (Figure 9). Similarly, psychiatric projects related to regenerative medicine products remained at approximately 2%-3% of the total, with the target conditions including dementia, developmental disorders, and schizophrenia (Figure 9). Conversely, psychiatric projects related to medical devices exhibited a notable increase, rising from 6.7% to 13.5% of the total projects related to medical devices from FY2020 to FY2022. Additionally, an upward trajectory was observed in target conditions, including dementia, mood disorders, and developmental disorders (Figure 9). Furthermore, the field of psychiatry was distinguished by a considerable proportion of cases falling within the “not applicable” category in the classification of Pharmaceutical and Medical Device Act, representing approximately 11%-15% of the total (Figure 9). This encompassed the development of programs centered on cognitive-behavioral therapy. Although psychiatry constituted only 8%-9% of the total number of AMED-funded projects, it accounted for 13.5% of all medical device projects in FY2022. This indicates that the number of projects seeking regulatory approval for medical devices has increased at a more pronounced rate in psychiatry than in other disease areas in recent years. The following factors may be associated with this increase in the number of medical device projects; i.e., despite the diverse and vast body of knowledge about psychiatric disorders (from genetics and molecular biology to cognitive and behavioral science), the etiology remains unknown, and digital technologies, including artificial intelligence (AI), is attracting significant attention in the field of medical device for psychiatric disorders as a modality that can break through these obstacles.

**Figure 8.**
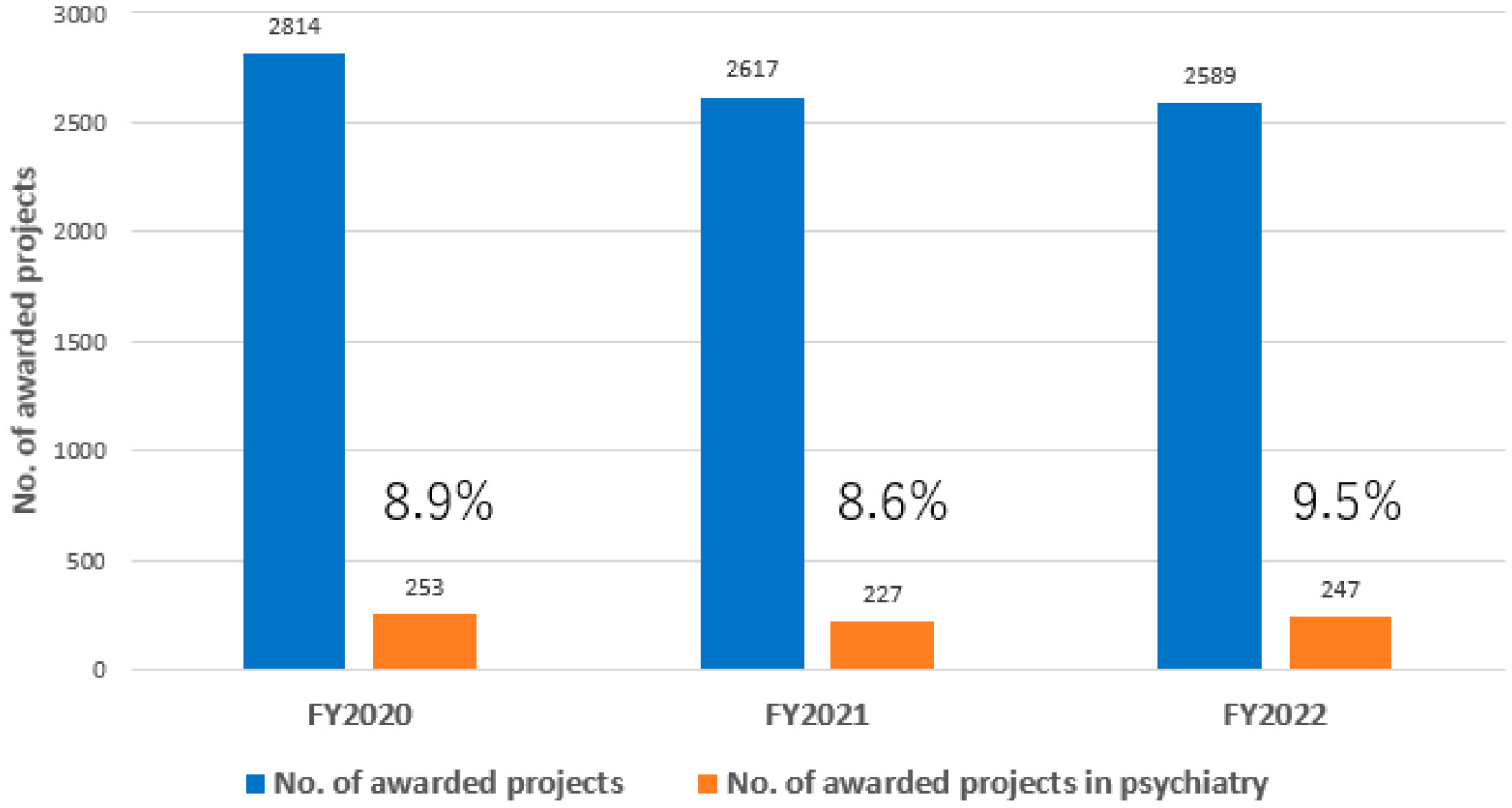
Trends in number of awarded projects related to psychiatry in FY2020-2022. The present figure was based on the ICD-10, and involved the selection of projects whose primary or secondary target diseases were psychiatric disorders. FY: fiscal year; ICD-10: International Classification of Diseases, 10th Revision.

**Figure 9.**
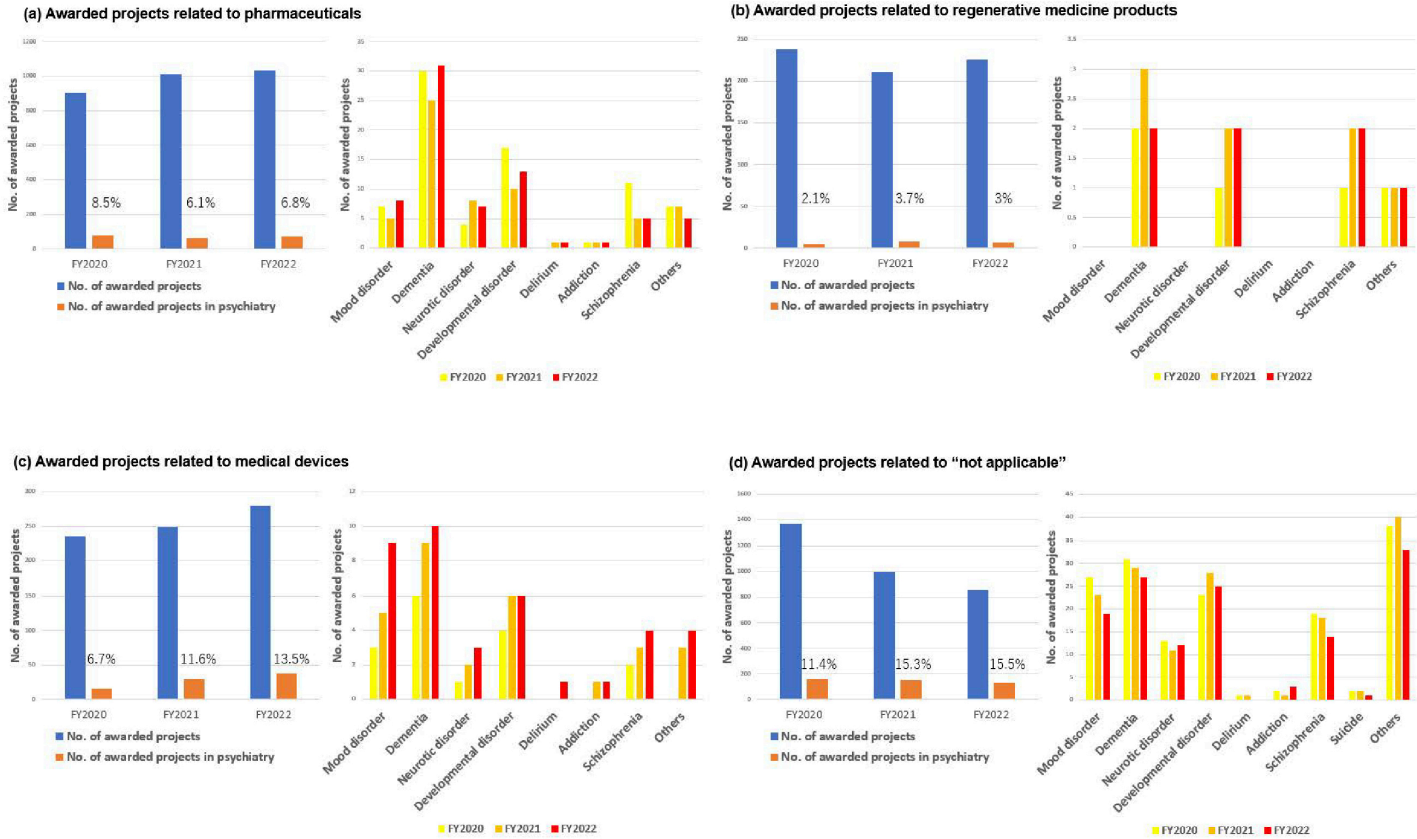
(Left) Trends in number of awarded projects related to classification of the Pharmaceutical and Medical Device Act in FY2020-2022, (right) trends of target diseases related to the classification in psychiatry. The classification of the Pharmaceutical and Medical Device Act is as follows; (a) Awarded projects related to pharmaceuticals; (b) Awarded projects related to regenerative medicine products; (c) Awarded projects related to medical devices; (d) Awarded projects related to “not applicable” category; There is a degree of overlap in the target diseases and drug classifications addressed in some projects. FY: fiscal year.

The proportion of digital mental health projects related to AI and telemedicine constituted between 18 and 24% of all psychiatric projects over the period FY2020-2022. This represents a notable trend within the broader psychiatric landscape (Figure 10). Specifically, there was the development of Software as a Medical Device that could screen for depression and assess its severity by collecting biometric and activity data through a wearable device and analyzing that data using machine learning algorithms ^(12)^. Furthermore, applications were developed with the objective of reducing the fear of cancer recurrence among breast cancer survivors through the use of smartphone psychotherapy ^(13)^. The project employed a decentralized clinical trial in which patients could participate remotely via their smartphones. The cognitive-behavioral therapy app offers a vision of the future in which patients can receive pain-relieving medical care at any time and in any location, obviating the need for hospital visits. In this respect, it is an achievement that overcomes the unmet needs of patients through digital mental health. The advancement of digital mental health technologies has emerged as a dominant trend in psychiatric R&D in recent years.

**Figure 10.**
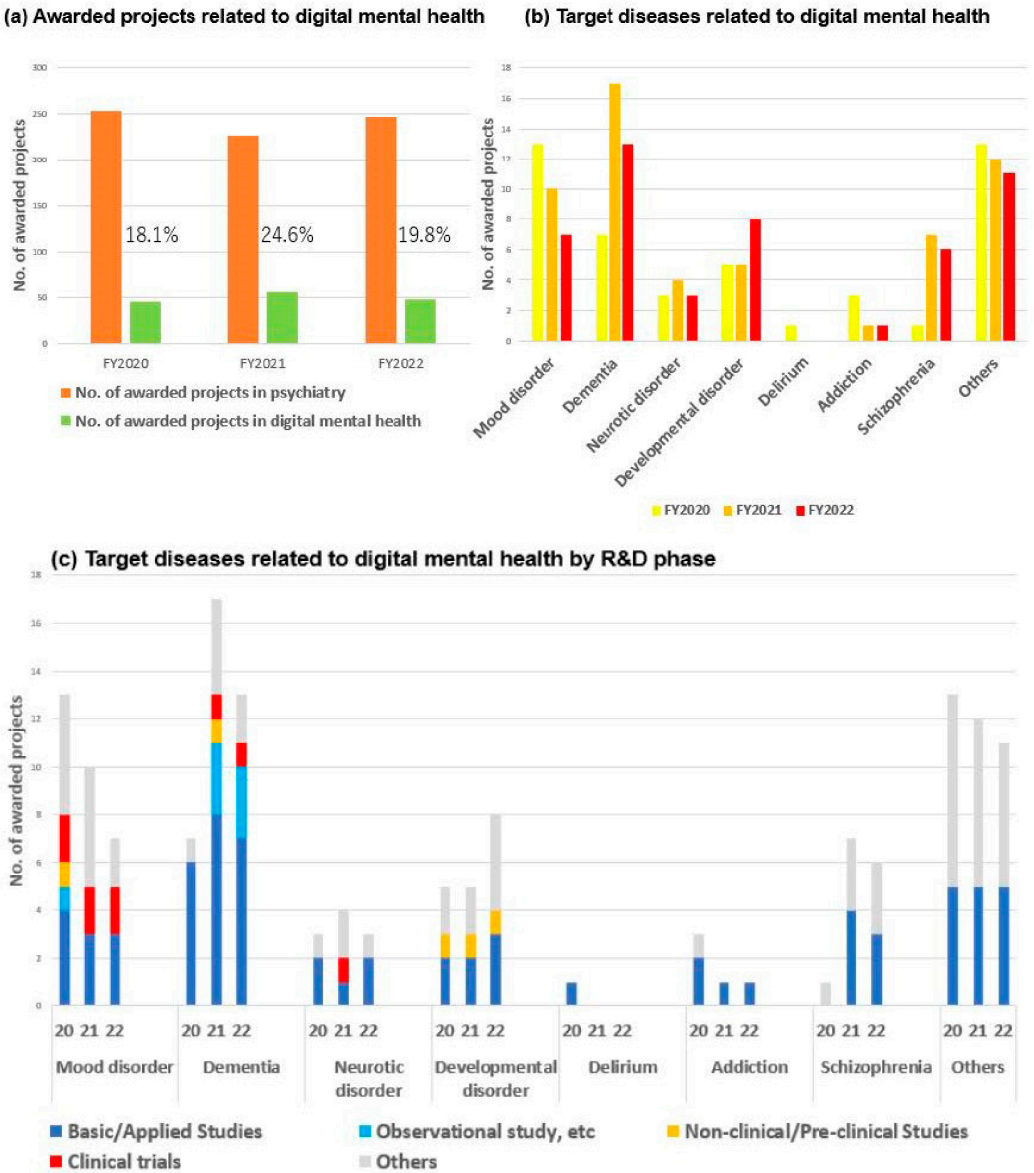
Trends of awarded projects related to digital mental health in FY2020-2022. (a) Trends in number of awarded projects related to digital mental health in FY2020-2022; (b) Trends of target diseases related to digital mental health; (c) Trends of target diseases related to digital mental health by R&D phase. There is a degree of overlap in the target diseases and drug classifications addressed in some projects. FY: fiscal year; R&D: Research and Development.

It was evident that there was a preference for obtaining regulatory approval for digital mental health projects designed to address mood disorders. Digital mental health projects constituted a significant proportion of psychiatry, with dementia, developmental disorders, and mood disorders representing the most prevalent psychiatric disorders addressed (Figure 10). Upon initial examination, however, the data appeared to contradict these trends. Figure 10b depicts a decline in the number of projects targeting mood disorders and dementia. This discrepancy can be resolved by conducting a review of the R&D phase for each target disease. In other words, in mood disorders and dementia, where the number of projects was declining, the R&D phase advanced to the clinical trial stage from the basic/applied and preclinical stages (Figure 10). With respect to projects that advanced to the clinical trial phase, digital technology and AI were no longer viewed as development items but rather as foundational technologies. Therefore, the apparent decline in the number of projects for mood disorders and dementia can be attributed to their removal from the development items in the aforementioned projects.

Further efforts need to be made in terms of funding agencies to overcome psychiatric disorders. AMED’s R&D funding for psychiatric disorders is 16.2 billion JPY in FY2023 ^(14)^. On the other hand, the National Institute of Mental Health (NIMH) funding alone was about 2.3 billion U.S. dollars in FY2023, which is more than the entire AMED budget ^(15)^. Although the AMED’s budget itself is small compared to the NIMH, and the amount of funding for psychiatric disorders is limited, the promotion of collaboration between industry, academia, and government in the field of medical devices utilizing AI and other cutting-edge technologies, e.g., optimizing the matching scheme between companies and academia, and R&D for disease-specific social and unmet medical needs, is expected to contribute to overcoming psychiatric disorders. In this context, digital mental health approaches are expected to become increasingly important.

This paper outlines the characteristics of projects included in AMED’s first and second mid- and long-term plans. In recent years, there has been a notable increase in the number of projects pertaining to the use of medical devices in the field of psychiatry, particularly within the domain of digital mental health. It is anticipated that subsequent research will be conducted with the objective of obtaining regulatory approval under the Pharmaceutical and Medical Device Act.

## Article Information

### Conflicts of Interest

None

### Acknowledgement

We would like to thank Mrs./Drs. Takeo Asano, Osamu Kato, Seiya Hirakawa, Mamoru Kaneko, Tetsushi Kagawa, and Ms. Yukiko Moriyasu at the AMED for supporting our research activity.

### Author Contributions

YY and YM designed the concept. YY wrote the first draft of the manuscript. YM and MH reviewed it and provided critical revisions. All authors approved the final version of the manuscript.

## Supplement

Supplementary Figures
