# Supplementary material for: Funding Trends in Japan Agency for Medical Research and Development (AMED): Focus on Psychiatry
Source: JMA J. 2025 Apr 4;8(2):385–94. doi: 10.31662/jmaj.2024-0391 (PMC12095502; doi:10.31662/jmaj.2024-0391)
Supplement: Supplementary Figures [file 2433-3298-8-2-0385-s001.pdf]

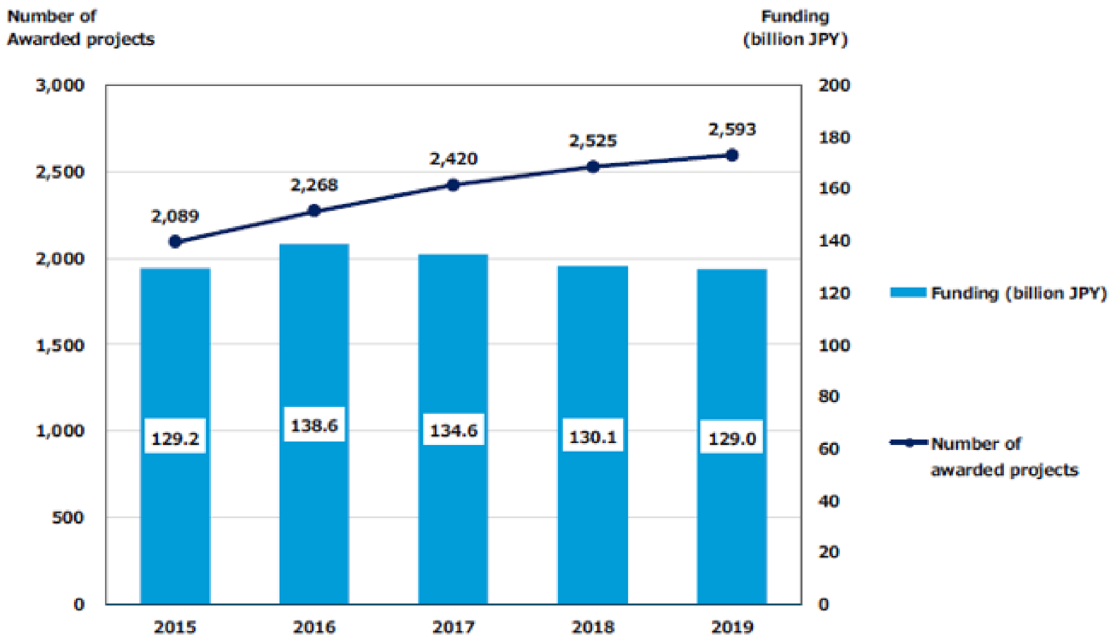

Supplementary Figure 1. Number of awarded projects and amount of funding in FY2015-2019 (adapted with permission from AMED data book: first medium- to long-term plan)[(10)]. The total number of awarded projects and the aggregate funding in JPY represent the sum of all projects funded in each FY. The figures represent the cumulative sum of all awarded projects, encompassing both newly awarded projects and those that were continued from the previous year.

FY: fiscal year; JPY: Japanese yen.

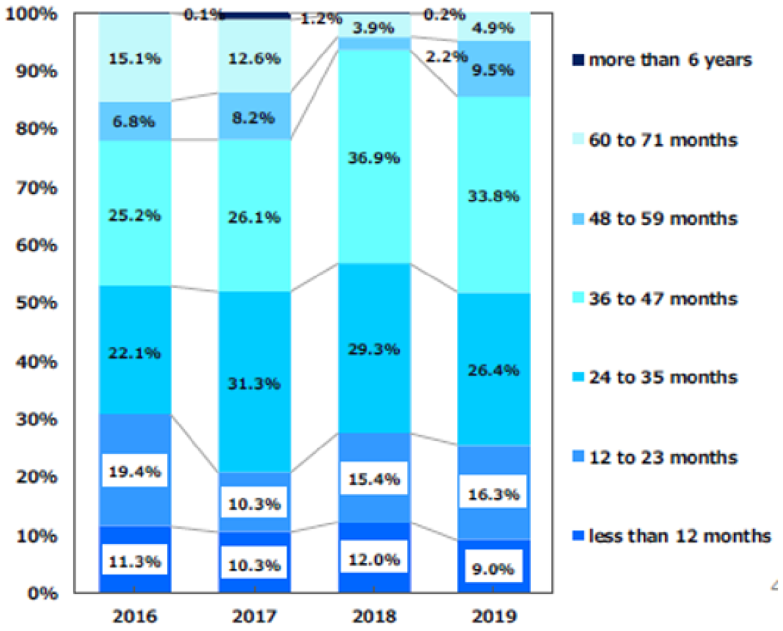

Supplementary Figure 2. Percentage of newly awarded projects by research duration in FY2016-2019 (adapted with permission from AMED data book: first medium- to long-term plan)[(10)]. The percentages presented are relative to the total number of awarded projects (100%) in each FY.

FY, fiscal year.

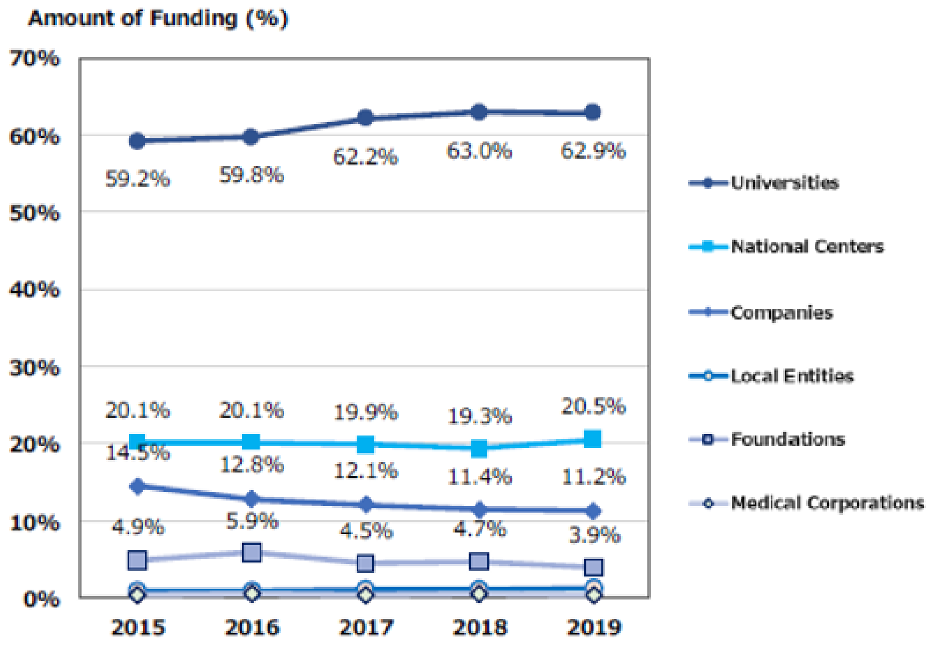

Supplementary Figure 3. Amount of funding by the type of institutions in FY2015-2019 (adapted with permission from AMED data book: first medium- to long-term plan)[(10)]. The term "amount of funding" denotes the total sum of financial resources received by each institution.

FY: fiscal year.

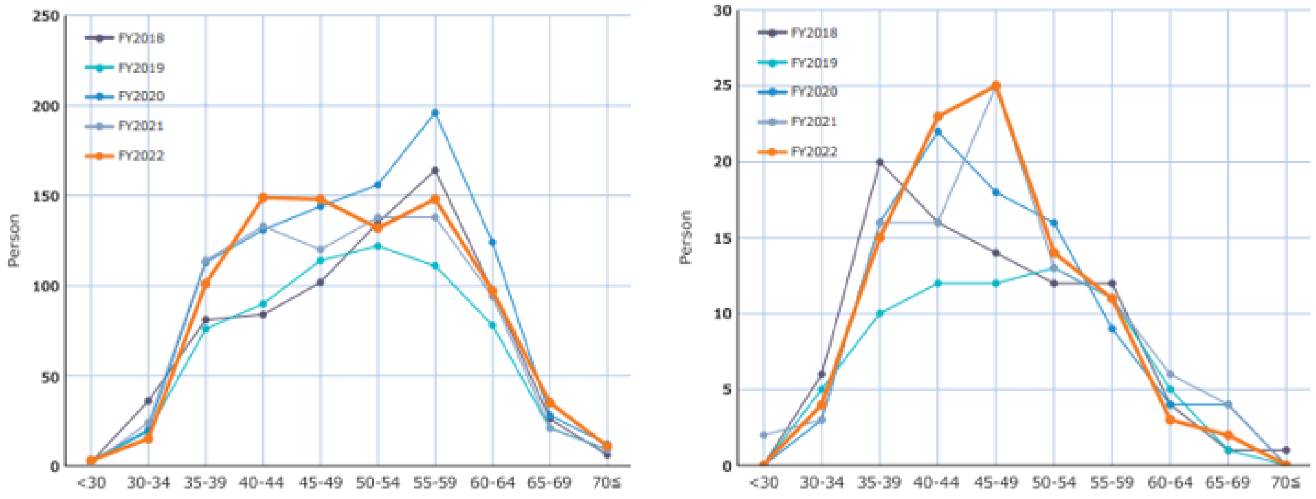

Supplementary Figure 4. Trends in the average age of male (left) and female (right) PIs by age group in FY2018-2022 (adapted with permission from AMED DataBook 2022)[(11)]. They are calculated by age group based on the number of PIs which is the aggregate number for the newly awarded projects in each FY, and their ages are as of the start of each FY of research launch based on their birth dates. This figure is derived from the Cross-ministerial R&D Management System (e-Rad) (as of October 2023).

FY: fiscal year; PI: principal investigator; R&D: Research and Development.
